# Development of PEO in Low-Temperature Ternary Nitrate Molten Salt on Ti6Al4V

**DOI:** 10.3390/ma18153603

**Published:** 2025-07-31

**Authors:** Michael Garashchenko, Yuliy Yuferov, Konstantin Borodianskiy

**Affiliations:** Department of Chemical Engineering, Ariel University, Ariel 40700, Israel; mihail.garashch@msmail.ariel.ac.il (M.G.); yuliyy@ariel.ac.il (Y.Y.)

**Keywords:** Ti alloy, plasma electrolytic oxidation, titanium oxide, ternary molten salt, calcium phosphates, corrosion resistance, biocompatibility

## Abstract

Titanium alloys are frequently subjected to surface treatments to enhance their biocompatibility and corrosion resistance in biological environments. Plasma electrolytic oxidation (PEO) is an environmentally friendly electrochemical technique capable of forming oxide layers characterized by high corrosion resistance, biocompatibility, and strong adhesion to the substrate. In this study, the PEO process was performed using a low-melting-point ternary eutectic electrolyte composed of Ca(NO_3_)_2_–NaNO_3_–KNO_3_ (41–17–42 wt.%) with the addition of ammonium dihydrogen phosphate (ADP). The use of this electrolyte system enables a reduction in the operating temperature from 280 to 160 °C. The effects of applied voltage from 200 to 400V, current frequency from 50 to 1000 Hz, and ADP concentrations of 0.1, 0.5, 1, 2, and 5 wt.% on the growth of titanium oxide composite coatings on a Ti-6Al-4V substrate were investigated. The incorporation of Ca and P was confirmed by phase and chemical composition analysis, while scanning electron microscopy (SEM) revealed a porous surface morphology typical of PEO coatings. Corrosion resistance in Hank’s solution, evaluated via Tafel plot fitting of potentiodynamic polarization curves, demonstrated a substantial improvement in electrochemical performance of the PEO-treated samples. The corrosion current decreased from 552 to 219 nA/cm^2^, and the corrosion potential shifted from −102 to 793 mV vs. the Reference Hydrogen Electrode (RHE) compared to the uncoated alloy. These findings indicate optimal PEO processing parameters for producing composite oxide coatings on Ti-6Al-4V alloy surfaces with enhanced corrosion resistance and potential bioactivity, which are attributed to the incorporation of Ca and P into the coating structure.

## 1. Introduction

Titanium and its alloys, particularly Ti-6Al-4V, are widely utilized in aerospace, medical, energy, and marine industries owing to their excellent corrosion resistance, high specific strength, thermal stability, and bio-inertness. Despite ongoing discussions regarding the potential long-term cytotoxicity of vanadium and aluminum, Ti-6Al-4V is still extensively used in both medical and aerospace fields [1,2,3,4,5,6,7,8].

Although titanium alloys possess excellent intrinsic properties, surface modification is often necessary to further improve attributes such as biocompatibility, corrosion resistance, and photocatalytic properties [3,9,10,11]. Among the available techniques, plasma electrolytic oxidation (PEO) has shown particular promise [12,13,14]. PEO is an electrochemical process used to produce an oxide coating on metal surfaces of valve metals such as aluminum, titanium, niobium, and zirconium, which develop thin and compact oxide layers. Although it has a similarity to electrochemical anodization [15,16,17,18], PEO operates at significantly higher voltages, which cause dielectric breakdown of the oxide layer, leading to micro-arc discharges. These discharges generate localized high temperatures and pressures within discharge channels, which drive phase transformations in the growing oxide layer [9,14,19,20,21].

Formed titanium dioxide commonly crystallizes in two primary polymorphs: anatase and rutile. During the initial stages of PEO, anatase, a metastable tetragonal phase characterized by high photocatalytic activity, typically forms first [22,23]. As processing continues and the applied voltage increases, microdischarges are initiated, generating localized high temperatures and pressures that induce a localized phase transformation of anatase to rutile above 600 °C [14,24,25]. Rutile, as the thermodynamically stable phase, exhibits greater hardness, corrosion resistance, and better wear resistance relative to anatase [26,27,28]. These phases have demonstrated high biocompatibility [9,29,30,31]. However, their individual contribution to biological performance remains debated [32]. Some studies, such as those by Boonrawd et al., suggest that anatase and rutile show no significant differences in terms of promoting a biomimetic apatite formation, cell proliferation, or viability [33,34].

The PEO process is governed by multiple parameters that critically affect the formation and properties of the resulting oxide coating on the substrate surface [9,20,35,36,37,38]. Typically, PEO is performed in aqueous electrolytes. Various electrolyte compositions, such as silicates, nitrates, carbonates, and phosphates, have been extensively studied for their influence on coating morphology and composition [9,35,39,40]. Notably, electrolytes enriched with phosphorus and calcium have been shown to promote the formation of bioactive phases such as calcium phosphates and hydroxyapatites, thereby improving both biocompatibility and corrosion resistance [41,42,43]. Additionally, the current mode and electrical parameters, including frequency, voltage, and current density, have a substantial influence on the morphology and properties of oxide coatings [9,37,44,45,46]. Elevated applied voltages typically result in more intense micro-arc discharges, which facilitate the formation of a thicker and more compact oxide layer, improving corrosion resistance [47]. Furthermore, increased pulse frequencies have been correlated with the formation of denser coatings characterized by lower porosity and enhanced mechanical properties [44]. According to Vasilyeva et al., extended treatment durations lead to a notable increase in both the average pore diameter and the overall porosity of the oxide layer [48]. Moreover, the use of aqueous electrolytes in the PEO process is often accompanied by excessive heating, which can lead to boiling, partial decomposition of the electrolyte, and fluctuations in its concentration. These effects may result in the formation of suboptimal coatings, electrolyte contamination, and degradation of coating properties [49,50]. To address these limitations, the present study employed a molten salt electrolyte for the PEO process, as previously demonstrated in [41,49,51].

Previous studies employed a binary eutectic molten salt electrolyte (NaNO_3_–KNO_3_); however, the high processing temperature of 280 °C hindered the incorporation of desired additives [49,51,52]. To enable lower processing temperatures, this research utilized a ternary eutectic electrolyte composed of NaNO_3_–KNO_3_–Ca(NO_3_)_2_, with a melting point of 132 °C, previously discovered as effective for consistent PEO coating formation on Ti-6Al-7Nb alloys [41,53,54]. This lower temperature is advantageous for the incorporation of ammonium dihydrogen phosphate (ADP) during processing. However, the use of this ternary eutectic molten salt electrolyte for oxide layer formation on Ti-6Al-4V has not been previously reported. The incorporation of calcium phosphate compounds into the coating is known to enhance biocompatibility and bioactivity, making such coatings highly suitable for orthopedic and dental implants and broader prosthetic applications [7,8].

Thus, this study aimed to identify the optimal parameters for the formation of PEO coatings on Ti-6Al-4V substrates and comprehensively evaluate their morphology and performance. The surface morphology of the coatings was analyzed using scanning electron microscopy (SEM), while chemical and phase composition were examined via energy-dispersive X-ray spectroscopy (EDX) and phase analysis through X-ray diffraction (XRD). The wettability of formed surfaces was evaluated through contact angle (CA) measurements using Hank’s solution. Furthermore, the corrosion behavior of the coating was studied with potentiodynamic polarization (PDP) tests in a simulated body fluid (Hank’s solution).

## 2. Materials and Methods

### 2.1. Coating Fabrication

Rectangular samples of Ti-6Al-4V alloy (TVA, Al 6 wt.%, V 4 wt.%, bal. Ti, Testbourne Ltd., (Basingstoke, UK) with dimensions of 20 × 40 × 1 mm were used in this study. Prior to surface treatment, samples were polished using #600- and #1200-grit abrasive paper, followed by rinsing in distilled water, acetone, and ethanol, and finally, the samples were air-dried.

The PEO process was performed in a ternary eutectic molten salt electrolyte composed of 42 wt.% Ca(NO_3_)_2_, 16 wt.%, and 42 wt.% KNO_3_ (each with 99.0% purity, Carlo Erba Reagents GmbH, Emmendingen, Germany) at 160 °C, following a procedure described elsewhere [53,55]. The load of the crucible was 300 g of ternary molten salt electrolyte. Ammonium dihydrogen phosphate (ADP, NH_4_H_2_PO_4_ 99.9%, Supelco, Bellefonte, PA, USA) was introduced as a phosphorus-containing source in concentrations ranging from 0.1 to 5 wt.% under vigorous stirring at 140 °C. The PEO treatment was performed using an MP2-AS 35 (Magpuls, Sinzheim, Germany) power supply in an anodic unipolar square current with a 50% duty cycle. Applied voltages were set at 200, 300, or 400 V, and current frequencies were set at 50, 500, and 1000 Hz. Electrical parameters were monitored by a Fluke Scope-Meter 199 C (2.5 GS/s, Everett, WA, USA). The temperature ternary molten salt electrolyte was maintained using a custom-made furnace in a cylindrical Ni crucible (internal diameter of 4; height of 19 cm), which simultaneously served as the counter electrode.

### 2.2. Coating Characterization

Surface morphology and cross-sectional examinations were analyzed using a scanning electron microscope (SEM) with 6510 LV-JSM (Jeol Ltd., Tokyo, Japan). The elemental composition was determined using an energy-dispersive X-ray spectroscopy (EDX) system, Thermo Scientific Fisher EDX 7 (Thermo Fisher Scientific Inc., Waltham, MA, USA). The average elemental composition and the corresponding standard deviation using Student’s t-distribution were determined based on nine measurements, each taking over an area of at least 250 µm^2^ at two distinct zones of surface locations. Surface cross-sections were identified using SEM MAIA3 (TESCAN, Czech Republic) with an EDX system by Oxford Instruments (Abingdon, UK).

Phase identification was conducted by X-ray diffraction (XRD, X’Pert Pro diffractometer, PANalytical B.V., Almelo, the Netherlands) with CuKα radiation (λ = 1.542 Å) over a 2θ range of 20–80°, with a step size of 0.02° and a rate of 1 s/step. This was operated at 40 kV and 40 mA at a grazing incident mode of 1°.

Surface wettability was assessed by measuring the contact angle (CA) using an RH-2000-3D digital microscopy system (Hirox Ltd., Tokyo, Japan) in sessile drop mode using a 10 μL drop of Hank’s solution (Sartorius, Göttingen, Germany, NaCl 138.00, glucose 5.60, KCl 5.33, CaCl_2_ 1.26, KH_2_PO_4_ 0.44, MgCl_2_-6H_2_O 0.50, MgSO_4_·7H_2_O 0.41, NaHCO_3_ 4.00, and Na_2_HPO_4_ 0.30 mM) at room temperature (25 °C), and CA was recorded after 15 min of droplet residence. The average CA value and standard deviation (based on Student’s t-distribution) were calculated based on ten measurements taken from different surface locations.

### 2.3. Corrosion Resistance Test

Corrosion resistance was examined using an Ivium XRe potentiostat (Ivium Technologies B.V., Eindhoven, the Netherlands) in the imitated biological environment, employing Hank’s solution (Sartorius, Göttingen, Germany) as the electrolyte in a three-electrode cell configuration. A Pt electrode served as a counter electrode (CE), and a Reference Hydrogen Electrode (Gaskatel, Kassel, Germany) served as a reference electrode (RE). The pH of Hank’s solution was monitored with an AZ 86,505 pH meter (AZ Instrument Corporation, Taichung, Taiwan). Prior to electrochemical measurements, all samples were immersed in Hank’s solution for 1–3 h to reach a steady state for the working electrode (WE). The potentio dynamic polarization (PDP) tests were conducted at a scanning rate of 1 mV/s, with a potential range of −250 mV to +250 mV vs. RE. The corrosion performance of the coatings was evaluated using a corrosion cell with a 6 mm diameter contact area. Owing to the small contact area, each specimen was tested at six different locations, allowing for statistical analysis of the results using the standard deviation calculated according to Student’s t-distribution.

Two sets of samples were investigated: The first series was produced using various concentrations of ADP additives in the low-melting-point ternary eutectic electrolyte; the second involved variations in the applied voltage and current frequency during the PEO process. The experimental conditions were designated using the format TVA {x; y; z}, where x denotes the weight percentage of the introduced ADP, y is the applied voltage, and z is the frequency of the PEO current: for example, TVA {0.1; 400; 50}.

## 3. Results and Discussions

### 3.1. PEO Processing

**Figure 1:**
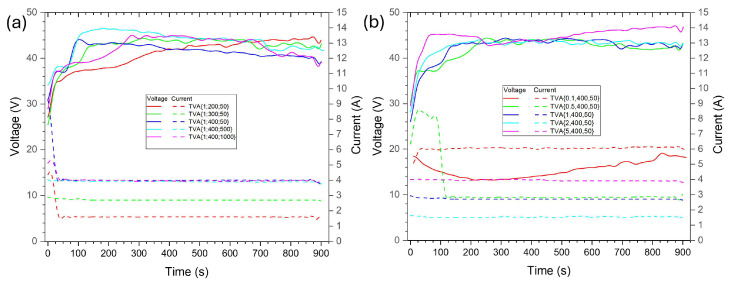
Current–time and voltage–time curves for the PEO process in ternary eutectic electrolyte: (**a**) series with varying voltage and frequency; (**b**) series with different ADP concentrations.

Figure 1 presents the corresponding voltage–time and current–time curves, showing that samples obtained in electrolytes containing ADP additives in concentrations ranging from 0.5 to 2 wt.% exhibit similar behavior. The observed trends in discharge activity and coating development align with the typical progression of the PEO process. This process can be divided into three main stages, each characterized by distinct voltage behavior. During the initial stage, the formation of a thin and compact dielectric barrier layer causes a rapid rise in voltage. The second stage is marked by a slower voltage increase, attributed to the onset of sparse micro-discharges as the oxide layer reaches the threshold for electrical breakdown. At this point, thermal stresses generated by discharge heating and rapid cooling induce localized cracking in the oxide layer. This facilitates plasma discharges, which propagate the development of an outer porous and dense barrier oxide layer. Upon reaching a critical voltage, the process enters the third stage, where micro-discharges evolve into more intense and stable arcs, leading to voltage stabilization and continued growth of the outer oxide layer, which corresponds to typical PEO behavior reported previously in [9,14,19,20,56,57].

The voltage–time curve response to the samples treated with 0.1 wt.% ADP, similarly to that of the sample without any ADP addition, does not exhibit features that are characteristic of the PEO process. This behavior is likely due to the formation of a very thin and dense passive oxide layer, which prevents dielectric breakdown. In contrast, the sample obtained in electrolytes with an addition of 5 wt.% ADP showed distinct surface coverage dominated by phosphate compounds. Its voltage–time curve revealed a fourth stage in addition to the three characteristic stages associated with the PEO process. It is assumed that during the initial three stages, a conventional PEO oxide coating is formed. Subsequently, the conjunction of soft spark discharges with electrophoretic deposition phenomena leads to a further voltage increase and the deposition of phosphate-rich phases on the coating’s surface [41].

The addition of ADP to the electrolyte results in the formation of reaction products that act as stabilizers, promoting the generation of softer microdischarges [42,58]. According to Bertuccioli et al., the incorporation of phosphate-containing compounds alters the spark temperature during the PEO process [59]. This behavior is likely associated with the formation of hard-melting and ionic phosphates within the electrolyte. Consequently, the phase composition and chemical characteristics of the coating are influenced by the spark temperature, electrolyte composition, and the nature of electrochemical transport [41].

### 3.2. Coating Characterization

**Figure 2:**
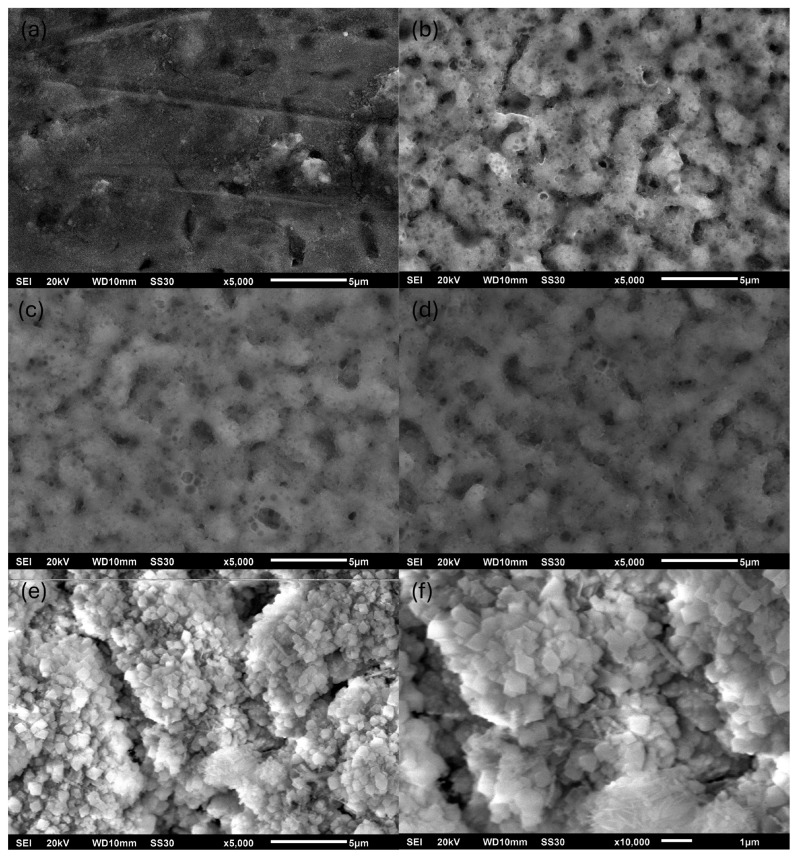
SEM images of surface morphologies obtained by PEO with varying ADP concentrations: (**a**) 0.1, (**b**) 0.5, (**c**) 1, (**d**) 2, (**e**) 5, and (**f**) 5 wt.% with higher magnification. All coatings were synthesized at 400 V and 50 Hz.

Figure 2 illustrates the surface morphology of the coatings synthesized at 400 V and 50 Hz with varying concentrations of the ADP additive. The coatings formed with 0.5, 1, and 2 wt.% ADP exhibit typical random porosity associated with the PEO process. In contrast, no coherent coating was observed on samples treated in the absence of ADP, as shown in Figure S1 (Supplementary Materials). The substrate’s surface underwent slight oxidation during the PEO process, resulting in a sponge-like surface morphology. The coating obtained with 0.1 wt.% ADP was extremely thin, likely due to a lack of phosphorus-containing compounds involved in the coating’s formation.

**Figure 3:**
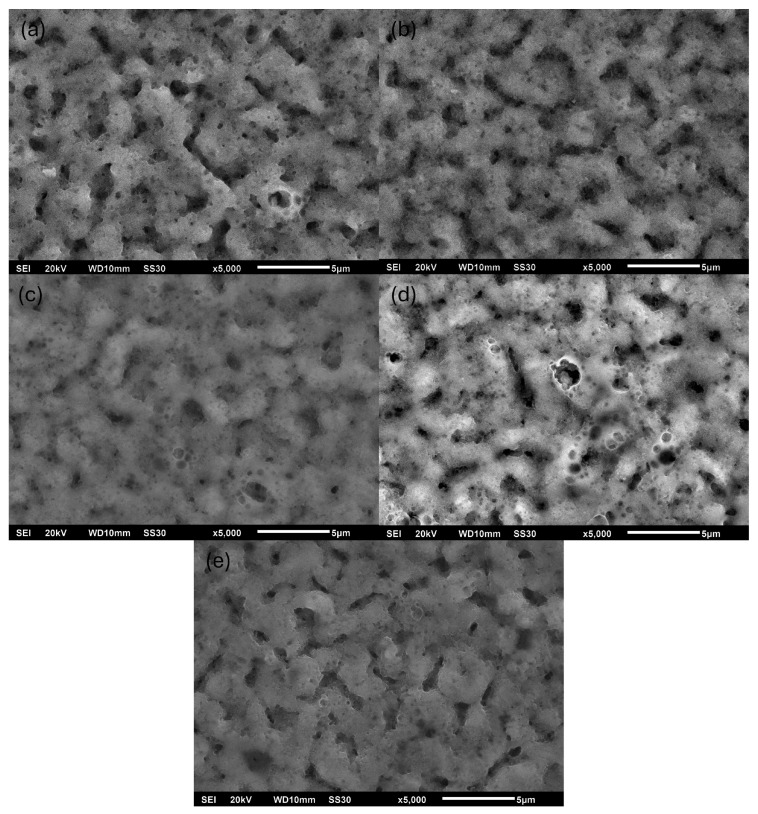
SEM images of surface morphologies obtained by PEO under varying electrical parameters: (**a**) 200 V/50 Hz; (**b**) 300 V/50 Hz; (**c**) 400 V/50 Hz; (**d**) 400 V/500 Hz; (**e**) 400 V/1000 Hz. All coatings were synthesized in molten electrolytes with 1 wt.% ADP.

At 5 wt.% ADP addition, the coating displayed notable porosity, which was accompanied by several particles. As shown in Figure 2e,f and Figure S2 (magnified view), these surfaces include rhombohedral-shaped particles on the surface and several needle-like particles. The EDX analysis suggested these morphologies correspond to calcium phosphate compounds. Figure 3 presents the influence of PEO electrical parameters with the addition of 1 wt.% ADP. It could be concluded that all samples demonstrate successful formation of typical PEO coatings characterized by surface porosity.

**Figure 4:**
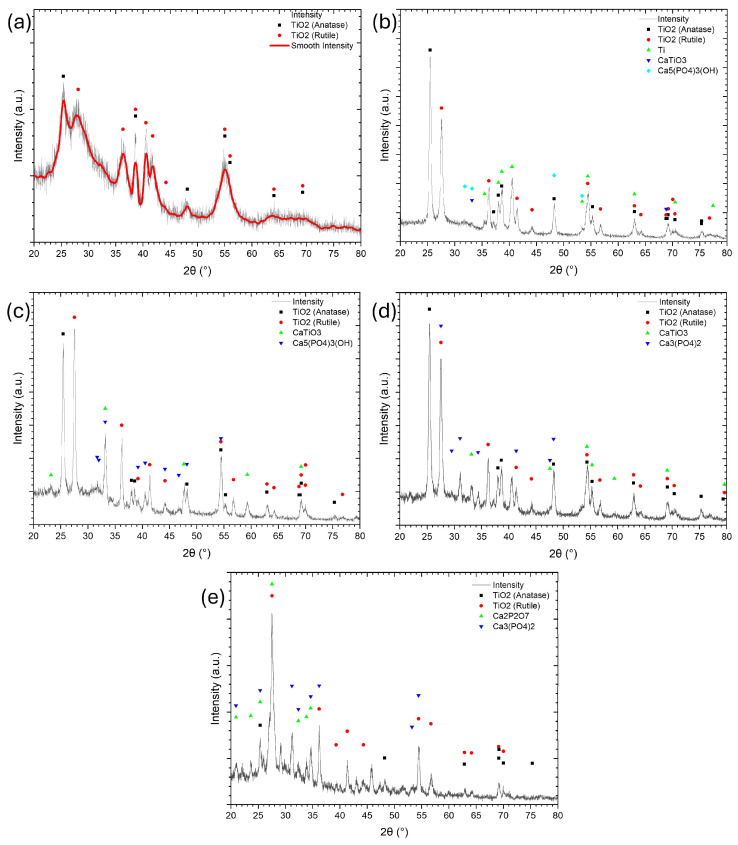
XRD patterns showing the phase composition of ceramic coatings obtained by PEO with varying ADP concentrations: (**a**) 0.1, (**b**) 0.5, (**c**) 1, (**d**) 2, and (**e**) 5 wt.%. All coatings were synthesized at 400 V and 50 Hz.

The phase composition of the PEO coatings was analyzed using XRD, and the corresponding patterns are presented in Figure 4. The analysis revealed that the predominant phases were anatase and rutile. In the coating obtained with 0.1 wt.% ADP, only titanium oxide phases were detected with strong amorphization, suggesting that the limited ADP content contributed to the incorporation of calcium phosphates in quantities too low to be detected by XRD. The XRD analysis revealed that the predominant crystalline phases were the anatase and rutile forms of titanium dioxide. The anatase phase exhibited prominent diffraction peaks at 2θ ≈ 25.36°, 37.05°, 37.91°, 48.16°, 54.05°, 55.20°, 62.87°, and 75.28°, corresponding to the (101), (103), (004), (200), (105), (211), (204), and (215) planes, respectively (ICSD No. 01-073-1764) [41,44,60,61]. The rutile phase was identified by characteristic peaks at 2θ ≈ 27.47°, 36.13°, 41.30°, 44.09°, 54.39°, 56.69°, 69.09°, and 69.92°, which correspond to the (110), (101), (111), (210), (211), (220), (301), and (112) planes, respectively (ICSD No. 01-073-1765) [9,60,61,62]. In contrast, coatings synthesized with 0.5, 1, and 2 wt.% ADP showed the presence of the perovskite-phase CaTiO_3_, which is attributed to the high local temperatures generated during the PEO process [21,37,57]. The increase in ADP concentration altered the phase composition of calcium phosphate compounds. At lower concentrations, hydroxyapatites (HAp) and calcium titanate phases were identified. However, as the ADP content in the molten salt electrolyte increased, calcium phosphate phases such as Ca_3_(PO_4_)_2_ and even calcium pyrophosphate appeared, particularly in the sample treated with 5 wt.% ADP. This behavior is likely related to variations in the initial Ca/P ratio in the ternary electrolyte system.

A higher ADP content reduces the Ca/P ratio, influencing the formation of specific phosphate phases. As noted by [63,64,65], the Ca/P ratio plays a critical role in determining the phase composition of calcium phosphate. The temperature effect also plays a significant role in phase evolution. As the ADP content increases, so does the electrolyte’s temperature, enhancing the stepwise decomposition of ADP and leading to the appearance of H_3_PO_4_ and trace quantities of water. The ADP decomposition begins at approximately 190 °C and proceeds according to the following reaction pathway reported in the literature [66,67,68]. This decomposition may lead to the formation of calcium phosphate compounds within the electrolyte, which can subsequently be incorporated into the coating during the PEO process:

(1) $$NH_{4}H_{2}PO_{4}\overset{190\;{}^{\circ}C}{\to }H_{3}PO_{4}+NH_{3},$$

(2) $$2H_{3}PO_{4}\overset{210\;{}^{\circ}C}{\to }H_{4}P_{2}O_{7}+H_{2}O,$$

(3) $$H_{4}P_{2}O_{7}\overset{350\;{}^{\circ}C}{\to }HPO_{3}+H_{2}O,$$

At 1 wt.% ADP, more intense HAp peaks were observed, indicating higher HAp contents compared to the 0.5 wt.% sample. Furthermore, the elevated ADP concentrations may intensify spark energy, promoting the formation of high-temperature phases such as tricalcium phosphate, which typically forms above 1200 °C, and this was detected in the coatings produced with 2 and 5 wt.% ADP.

**Figure 5:**
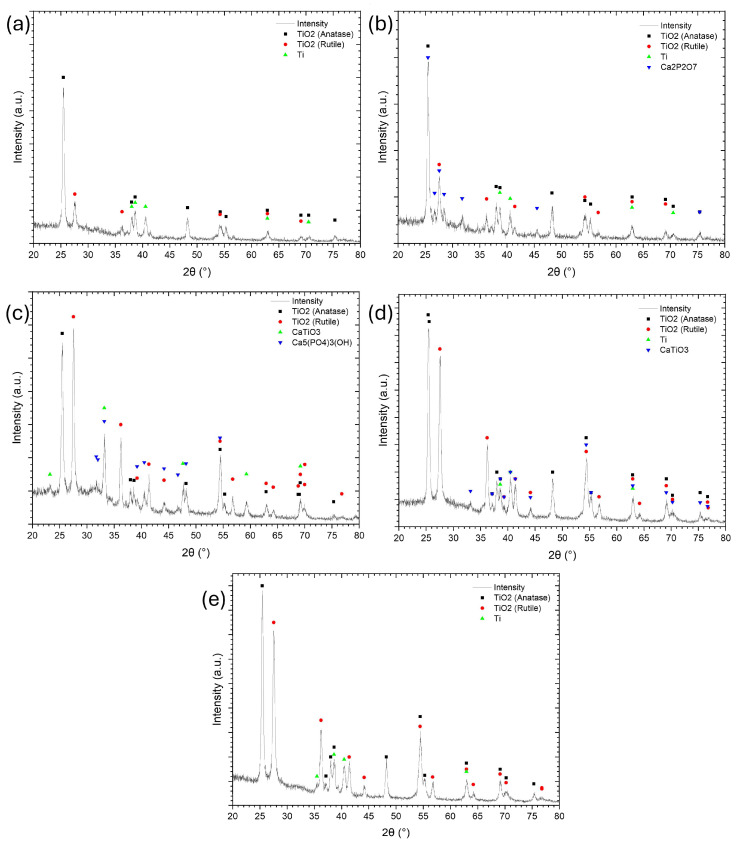
XRD patterns showing the phase composition of ceramic coatings obtained by PEO under different electrical parameters: (**a**) 200 V/50 Hz; (**b**) 300 V/50 Hz; (**c**) 400 V/50 Hz; (**d**) 400 V/500 Hz; (**e**) 400 V/1000 Hz. All coatings were synthesized in molten electrolytes with 1 wt.% ADP.

Figure 5 presents the XRD patterns for the coating series obtained under varying electrical parameters (voltage and frequency).

The main phases across all samples are titanium oxides—anatase and rutile. In the TVA {1; 200; 50} sample, the applied voltage during PEO was insufficient for elevating the electrolyte temperature to a level required for significant H_3_PO_4_ decomposition. Consequently, the XRD analysis did not reveal the presence of calcium phosphate phases. However, high temperatures generated in the discharge zones, followed by rapid cooling, created sharp thermal gradients. These conditions may promote the formation of intermediate calcium phosphate compounds, such as amorphous calcium phosphate, octacalcium phosphate, monocalcium phosphate, and dicalcium phosphate [69,70,71,72]. These compounds typically exhibit low crystallinity or are in their amorphous phase, making them difficult to detect by XRD. At 300 V–TVA {1; 300; 50}, the appearance of more intense sparks likely enables the formation of calcium pyrophosphate, a phase that crystallizes around 700 °C y. In the sample processed at 400 V, both HAp and calcium titanate phases were detected. The crystallization of HA is associated with temperatures around 1000 °C [73]. Additionally, the presence of the perovskite-phase CaTiO_3_ was observed, consistent with the high local temperatures generated during the PEO [21,37]. As frequency increases, the energy of individual discharges, as well as their thermal impact on the coating, tends to decrease. However, the number of discharges rises, leading to overall heating of the electrolyte and sample surface rather than remelting near the discharge channels. This likely accounts for the absence of crystalline calcium phosphate phases at higher frequencies.

**Figure 6:**
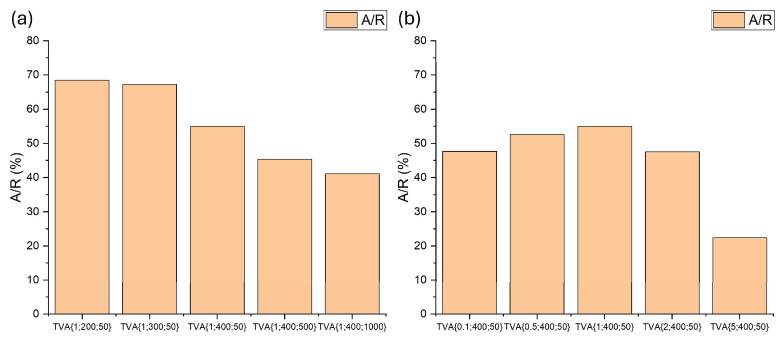
The anatase-to-rutile (A/R) ratio of PEO ceramic coatings: (**a**) synthesized with 1 wt.% ADP under varying voltages and frequencies; (**b**) formed with different ADP contents with fixed electrical parameters of 400 V and 50 Hz.

The primary phases formed during the PEO process, as identified by XRD analysis, belong to titanium oxide. The anatase phase is dominant, with the main (101) plane (ICSD 01-073-1764) appearing at 2θ ≈ 25.36°, exhibiting the highest relative intensity (100%). The main peak of the rutile phase is observed at 2θ ≈ 27.46°, corresponding to the (110) plane of rutile (ICSD 01-073-1765), and it appears with high intensities in most patterns. The anatase to rutile ratio can be estimated based on the intensity of their principal diffraction peaks using Equation (4), as described in reference [74]:

(4) $$f_{A/R}=1/(1+1.26\cdot \frac{I_{R(110)}}{I_{A}(101)})\%,$$

where I_R_ is the intensity of the (110) rutile reflection, and I_A_ is the intensity of the (101) anatase reflection. The calculated phase ratios are presented in Figure 6.

The data in Figure 6a demonstrates an inverse relationship between the A/R ratio and the applied voltage/frequency. A similar trend was reported by Saji, who observed a decrease in the A/R ratio with increasing voltage [75]. Elevated voltages intensify the micro-discharge activity on the titanium surface, resulting in higher local temperatures within the discharge channels, which favor the crystallization of the rutile phase. Saji also noted a reduction in the A/R ratio with increasing frequency [75]. This behavior may be attributed to a higher number of discharges with lower individual energy. The frequent discharge events hinder effective cooling between pulses, thereby promoting the anatase-to-rutile phase transformation through the combined effects of total electrolyte heating and frequent inputs from the discharges. Figure 6b shows that the A/R ratio initially increases with ADP additions up to 1 wt.%, followed by a decline at higher concentrations. A similar behavior has been reported by Rescigno in their studies [11]. This trend can be explained by the role of phosphorus-containing compounds, which at low concentrations may dope the titanium oxide, thereby inhibiting phase transition from the anatase phase to rutile, as previously reported in a review about the inhibition and promotion of titanium oxide phase transition [76]. However, at higher concentrations, these additives alter electrolyte conductivity, increasing discharge intensity and surface overheating, conditions that facilitate the transformation of anatase into rutile [67,77].

**Figure 7:**
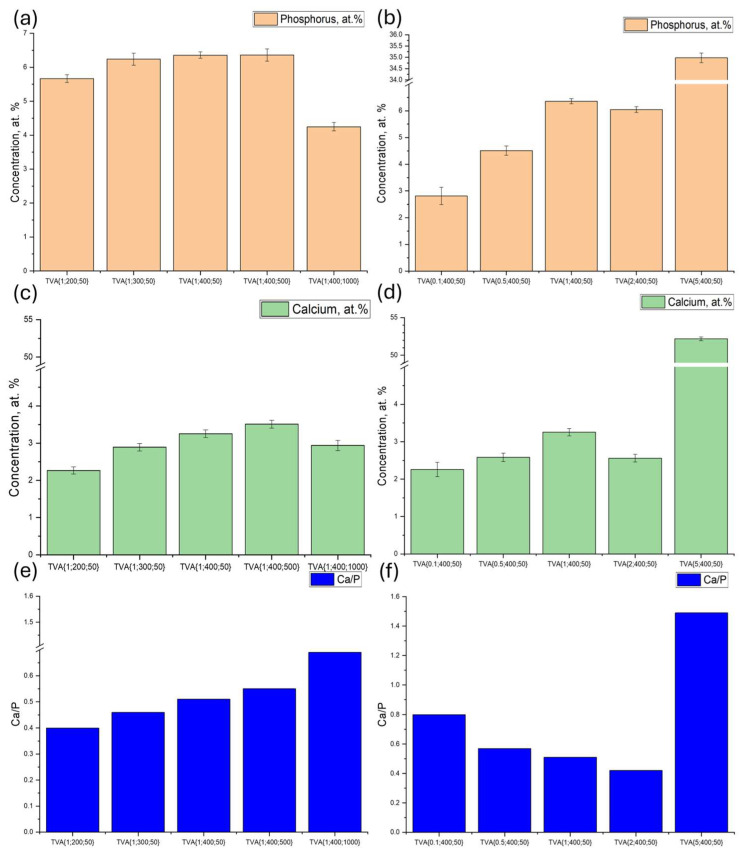
Elemental composition of Ca, P, and Ca/P ratio in the ceramic coatings obtained by PEO with the following: (**a**,**c**,**e**) an addition of 1 wt% ADP under varying voltages and frequencies; (**b**,**d**,**f**) different ADP contents under electrical parameters of 400 V and 50 Hz.

Figure 7a–f compare the atomic concentrations of Ca and P, as well as their Ca/P ratio. Both Ca and P contents increase with ADP concentrations up to 2 wt.%, followed by a slight decrease, and this is likely due to the high discharge energy, which may facilitate the penetration of Ca and P into deeper coating layers, making them less detectable by surface-sensitive techniques. The Ca/P ratio decreases up to 2 wt.% and then sharply increases at 5 wt.%, reflecting the preferential formation of stable Ca-rich compounds.

Increasing the voltage promotes Ca and P incorporation due to the higher discharge energy, while frequency has a nonlinear effect: minimal influences of up to 500 Hz, followed by a notable reduction at 1000 Hz. This behavior is likely due to a shorter discharge at higher frequencies, which limits Ca and P incorporation [78,79]. Nevertheless, the Ca/P ratio increases with both voltage and frequency, benefiting coating doping during the PEO process. Most intermediate calcium phosphate compounds decompose at temperatures between 200 °C and 800 °C, forming tri-calcium phosphate. HAp is more stable, with dehydration beginning at 600–900 °C and decomposition beginning at around 1200 °C, while tricalcium phosphate remains stable up to approximately 1300 °C [80,81]. The high local temperatures and rapid quenching promote the formation of amorphous phases in the PEO process, which are not detectable by XRD. Additionally, calcium ions can form calcium titanate, which is fused into the coating. Consequently, even when overall phosphate and calcium contents are reduced, the Ca/P ratio may increase due to the preferential formation of more thermally stable calcium-containing phases. A summary of the chemical composition of coatings determined by EDX analysis is presented in Table 1.

Cross-sectional SEM images and EDX elemental mapping of the coatings obtained with varying ADP concentrations and different current parameters are presented in Figures S3 and S4, respectively. The coating formed with 0.1 wt.% ADP exhibited a thickness of less than 0.6 ± 0.1 µm, which, based on surface morphology observations, indicates the formation of a non-PEO-type layer. In contrast, coatings produced with 0.5, 1, and 2 wt.% ADP showed comparable thicknesses in the range of 1.5–2.5 µm, characteristic of typical PEO coatings. Notably, the coating thickness of the TVA {5; 400; 50} sample reached approximately 45 ± 5 µm, which is attributed to the extensive precipitation of calcium phosphate compounds on the substrate’s surface. The variation in current parameters during the PEO process did not result in significant changes in the coating’s thickness. This behavior is associated with the stochastic nature of dielectric breakdown events that govern oxide layer growth in the PEO process. EDX mapping confirmed a uniform distribution of key elements such as Al, P, Ca, Ti, and V throughout the bulk of the PEO coatings, indicating their homogeneous incorporation.

It can be concluded that vanadium and aluminum were incorporated into the PEO coating in a relatively consistent ratio across most samples. An exception was observed for sample TVA {0.1; 400; 50}, which demonstrates a higher concentration of aluminum and vanadium due to the failure of proper PEO coating formation, as mentioned earlier. Additionally, the sample TVA {5; 400; 50} exhibits an almost fully covered Ca/P surface, as supported by SEM morphology and cross-sectional EDX mapping.

**Figure 8:**
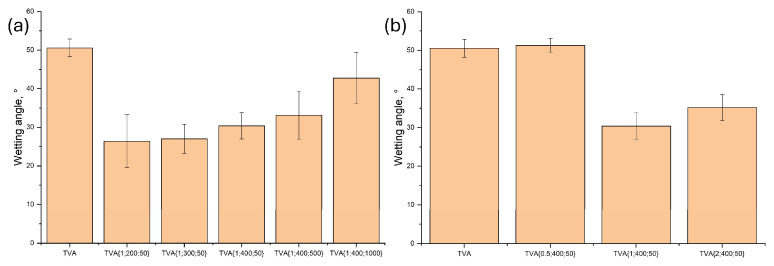
The contact angle of the ceramic coatings obtained by PEO with the following: (**a**) an addition of 1 wt.% of ADP under varying voltages and frequencies and (**b**) different ADP contents under electrical parameters of 400 V and 50 Hz.

The wettability of the synthesized ceramic surfaces was evaluated by measuring the contact angle with a biological medium (Hank’s solution), and the results are presented in Figure 8.

The uncoated Ti-6Al-4V alloy demonstrates high wettability and compatibility with biological environments, showing a contact angle of approximately 51°. According to studies by Mashtalyar et al., PEO coatings incorporating calcium phosphate compounds tend to display reduced contact angles, which may contribute to enhanced biocompatibility [29,82]. Contact angle measurements could not be conducted for samples treated with 0.1 and 5 wt.% ADP. In the case of 5 wt.% ADP, the surface was fully covered with phosphates, causing the complete spreading of the Hank’s solution droplet. For 0.1 wt.% ADP, the coating was too thin and non-uniform, also resulting in total droplet spreading. Coatings obtained with 1 and 2 wt.% ADP exhibited lower contact angles, particularly under lower voltages and frequencies, indicating improved wettability. This enhanced wettability facilitates better absorption of biological fluids, which is advantageous for biomedical applications [14,83].

### 3.3. Corrosion Behavior

**Figure 9:**
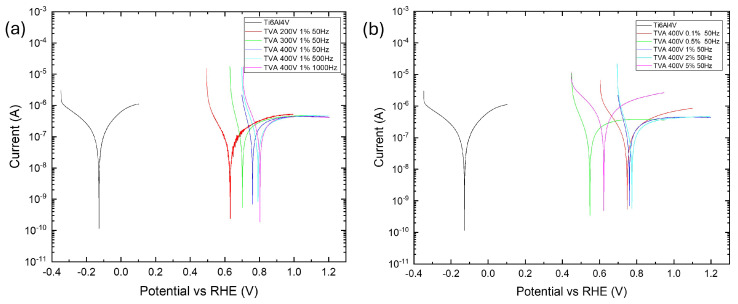
Corrosion behavior (Tafel plots) of ceramic coatings synthesized by PEO conducted in ternary eutectic electrolyte with the following: (**a**) an addition of 1 wt.% ADP under varying voltages and frequencies; (**b**) different ADP contents at fixed electrical parameters of 400 V and 50 Hz.

Potentiodynamic polarization (PDP) analyses were conducted using Hank’s solution to assess the corrosion behavior of the synthesized coatings under a simulated biological environment, as demonstrated in Figure 9. Owing to its chemical composition, which closely resembles that of human plasma, Hank’s solution is widely employed as an electrolyte for simulating a physiological environment. Its relatively high chloride ion concentration makes it more aggressive than other commonly used solutions, such as simulated body fluid, thereby providing a stringent environment for corrosion assessment [84,85,86]. The synthesized coatings exhibited elevated corrosion resistance in comparison with the original Ti-6Al-4V alloy.

The approximation of the Tafel region on polarization curves is a widely applied method for analyzing corrosion processes [41,87,88,89]. This approach relies on the linear relationship between the logarithm of the current density and the electrode potential, enabling the extraction of key electrochemical parameters. These include the open circuit potential (OCP) under steady-state conditions vs. RHE, corrosion potential (E_corr_) vs. RHE in Hank’s solution at a pH of 7.5 ± 0.1, corrosion current density (I_corr_), and the anodic and cathodic Tafel slopes (βa and βc) [41,87,88,89]. The values βa and βc were determined from the approximately linear region of the Tafel plot within ±25 to ±100 mV around E_corr_. The polarization resistance (Rp) was calculated using the Stern–Geary Equation (5), and the results are summarized in Table 2:

(5) $$R_{p}=\frac{\beta_{a}*\beta_{c}}{2.3*i_{corr}(\beta_{a}+\beta_{c})}$$

where β_a_ and β_c_ are the anodic and cathodic Tafel slopes, respectively; E_corr_ vs. RHE and i_corr_ are the corrosion potential and current density from Tafel plot fitting.

As shown in Figure 9, the corrosion-resistant Ti-6Al-4V alloy exhibits a strongly cathodic corrosion potential of −102 ± 52 mV vs. RHE with a high corrosion current density of 552 ± 19 nA/cm^2^. Anodic passivation current was 7.0 ± 8.2 µA/cm^2^, consistent with the rapid formation of an amorphous oxide coating, which corresponds to previously reported results [84,90,91]. Uncoated Ti alloys are prone to hydrogen embrittlement and pitting corrosion due to their cathodic potential and elevated passivation current, which facilitate hydrogen evolution and compromise galvanic compatibility in biological or chloride-containing environments. The resulting oxide coatings led to an anodic shift in corrosion potential and a reduction in passivation current, likely due to the decreased charge carrier mobility through the dielectric oxide layer. Coatings with 0.5, 1, and 2 wt.% ADP showed a pronounced anodic shift of corrosion potential to 525 ± 29, 793 ± 35, and 816 ± 70 mV vs. RHE, respectively, along with a uniform corrosion resistance of 0.076 ± 0.010, 0.066 ± 0.016, and 0.053 ± 0.010 MΩ·cm^2^. These enhancements are likely due to the enhanced incorporation of calcium and phosphate compounds into the coating, which mitigates the filling of voids and cracks formed in PEO. Furthermore, insoluble phosphate phases may also replace soluble species such as NH_4_VO_3_, KVO_3_, NaVO_3_, nitrates, or hydroxides, contributing to compactness and reducing layer defectivity [92,93,94]. The TVA {0.1; 400; 50} sample showed inconsistent corrosion values of 209 ± 81 nA/cm^2^ and high anodic passivation currents of 3.3 ± 0.4 µA/cm^2^, suggesting failed coating formation and high amorphization in comparison to anodic passivation currents of 1.3 ± 0.1, 1.9 ± 0.3, and 1.3 ± 0.5 µA/cm^2^ for 0.5, 1, and 2 wt.% of the ADP additive, respectively. Although TVA {0.5; 400; 50} exhibited good corrosion resistance, its lower anodic corrosion potential indicates diminished protection. It could be noted that I_corr_ exhibited a non-monotonic trend with increasing ADP concentrations. Specifically, the sample containing the lowest additive concentration showed an I_corr_ value of 209 ± 81 nA/cm^2^. Upon increasing the ADP content to 0.5 wt.%, a reduction in I_corr_ to 153 ± 24 nA/cm^2^ was observed, followed by a subsequent rise in Icorr with further increases in ADP content. A comparable trend was reported by Zhang et al., where the use of calcium hypophosphite as an electrolyte additive resulted in an I_corr_ of 12.7 nA/cm^2^ and decreased to 8.39 nA/cm^2^ at a higher concentration [95]. At 5 wt.% ADP, the corrosion current increased markedly due to an active corrosion process. This may be attributed to loosely structured heterogeneous coatings with numerous phosphate-rich inclusions, facilitating localized corrosion through charge carrier motion by grain boundaries and interfaces of various phases and particles. It should be noted that the Stern–Geary equation has certain limitations. These arise from the presence of anodic passivation currents and uncertainties in determining the cathodic and anodic Tafel slopes due to the pseudo-passive behavior of oxide films in low overvoltage regions. Such factors can affect the accuracy of Rp values, particularly in the case of complex, doped oxide coatings. Nevertheless, this method remains commonly applied for assessing the corrosion performance of titanium oxide coatings [9,41,84,86,87,88,89]. Based on the results, 1 and 2 wt.% ADP are the optimal content of the ADP additive for enhancing corrosion resistance.

Increasing voltage and frequency generally induced an anodic shift in corrosion potential from 658 ± 13 to 813 ± 10 mV vs. RHE, indicating improved corrosion resistance. However, samples {1; 200; 50} and {1; 300; 50} had lower anodic potentials of 658 ± 13 and 700 ± 3 mV vs. RHE, with comparable corrosion resistances throughout the sample series. At higher voltages {1; 400; **Z**}, intensified microdischarges likely promoted the formation of a denser barrier layer [9,96], thereby enhancing corrosion resistance. This effect is reflected in the corrosion potential (E_corr_) of the TVA {1; 400; 50} sample, which reached 793 ± 35 mV, consistent with the higher sparking and breakdown voltages characteristic of the coating. A similar trend was reported by Montazeri et al., where a maximum E_corr_ of 150 mV was observed for a sample treated via PEO at 400 V [36]. Higher frequencies offered no further benefits and complicated practical implementation. Therefore, TVA {1;400;50} could be considered an optimal PEO condition for corrosion protection parameters in a simulated biological environment.

## 4. Conclusions

In this study, composite PEO coatings were successfully synthesized on a Ti-6Al-4V alloy through the plasma electrolytic oxidation process in a ternary molten salt electrolyte with the study of the substitutional role of ADP additives.

This study focused on the synthesis of PEO coatings on TVA substrates using a ternary molten salt with ADP additives. The ADP concentration was found to play a critical role in the resulting coating performance. At 0.1 wt.%, the coating was thin and non-uniform, while concentrations of 0.5–2 wt.% produced uniform porous PEO coatings. An excessive content of ADP (5 wt.%) led to phosphate-based surface deposition of HAp and other calcium phosphate phases, which were confirmed by XRD analysis. The Ca/P ratio decreased with increasing ADP contents. Notably, at 5 wt.% ADP, the coating was completely covered by calcium phosphate compounds, which significantly decreased corrosion protection from 0.066 ± 0.016 at 2 wt.% ADP to 0.025 ± 0.011 MΩ·cm^2^ at 5 wt.% ADP.

The applied voltages and frequencies demonstrated an apparent effect on the resultant coating properties. The wettability tests carried out in Hank’s solution showed that increasing both the voltage and frequency elevated the contact angle values from 51 ± 4° for metal to 30 ± 4 for the {1; 400; 50} sample, indicating improved hydrophilicity in comparison with the uncoated metal, which may enhance the biological response of the surface. Simultaneously, changes in current parameters of the PEO process facilitated an increase in the Ca/P ratio and a concomitant decrease in the anatase-to-rutile ratio. Despite these changes, no significant differences in surface morphology were found. Finally, the sample {1; 400; 50} exhibited optimal anti-corrosion performance, as evidenced by a favorable corrosion potential of 956 ± 44 mV vs. RHE and a polarization resistance of 0.066 ± 0.016 MΩ·cm^2^ attributed to the combined influence of optimized PEO treatment parameters, electrolytes, and coating chemical and phase compositions.

Overall, ADP-assisted PEO significantly enhanced the corrosion resistance of Ti-6Al-4V, demonstrating its potential for protective and bioactive composite coatings in simulated biological environments.

## Figures and Tables

**Table 1:** Chemical composition of the obtained PEO ceramic surfaces.

Sample	Ti, at.%	Al, at.%	V, at.%	Ca, at.%	P, at.%
TVA {1, ***Y***, ***Z***}					
TVA {1; 200; 50}	80.1 ± 0.3	8.5 ± 0.2	3.4 ± 0.2	2.3 ± 0.2	5.7 ± 0.2
TVA {1; 300; 50}	79.0 ± 0.3	8.6 ± 0.1	3.3 ± 0.2	3.0 ± 0.2	6.2 ± 0.2
TVA {1; 400; 50}	78.8 ± 0.2	8.5 ± 0.2	3.3 ± 0.1	3.2 ± 0.2	6.3 ± 0.2
TVA {1; 400; 500}	78.6 ± 0.5	8.4 ± 0.2	3.2 ± 0.2	3.5 ± 0.1	6.4 ± 0.2
TVA {1; 400; 1000}	81.5 ± 0.3	8.2 ± 0.2	3.2 ± 0.2	2.9 ± 0.1	4.3 ± 0.1
TVA {***X***, 400, 50}					
TVA {0.1; 400; 50}	82.1 ± 0.2	9.4 ± 0.2	3.3 ± 0.3	2.3 ± 0.2	2.8 ± 0.3
TVA {0.5; 400; 50}	80.9 ± 0.3	8.8 ± 0.2	3.2 ± 0.2	2.6 ± 0.1	4.5 ± 0.2
TVA {1; 400; 50}	78.8 ± 0.2	8.5 ± 0.2	3.3 ± 0.1	3.2 ± 0.2	6.3 ± 0.2
TVA {2; 400; 50}	79.5 ± 0.3	8.5 ± 0.1	3.4 ± 0.2	2.6 ± 0.1	6.1 ± 0.1
TVA {5; 400; 50}	8.9 ± 0.1	0.5 ± 0.1	0.6 ± 0.1	52.2 ± 0.2	35.0 ± 0.2

**Table 2:** PDP results for ceramic coatings obtained by PEO in eutectic ternary electrolytes.

Sample	OCP [mV]	E_corr_ [mV]	I_corr_ [nA/cm^2^]	R_p_ [MΩ·cm^2^]	b_a_ [V/dec]	−b_c_ [V/dec]
Ti-6Al-4V	−77 ± 4	−102 ± 52	552 ± 19	0.083 ± 0.003	0.212 ± 0.016	0.211 ± 0.005
TVA {***X***, 400, 50}						
TVA {0.1; 400; 50}	832 ± 36	756 ± 36	209 ± 81	0.089 ± 0.027	0.082 ± 0.002	0.083 ± 0.013
TVA {0.5; 400; 50}	692 ± 54	525 ± 29	153 ± 24	0.076 ± 0.010	0.061 ± 0.021	0.049 ± 0.011
TVA {1; 400; 50}	956 ± 44	793 ± 35	219 ± 10	0.066 ± 0.016	0.090 ± 0.035	0.053 ± 0.011
TVA {2; 400; 50}	983 ± 34	816 ± 70	231 ± 55	0.053 ± 0.010	0.082 ± 0.024	0.044 ± 0.009
TVA {5; 400; 50}	734 ± 26	622 ± 5	653 ± 248	0.025 ± 0.011	0.076 ± 0.019	0.068 ± 0.006
TVA {1, ***Y***, ***Z***}						
TVA {1; 200; 50}	855 ± 40	658 ± 13	175 ± 12	0.070 ± 0.010	0.084 ± 0.005	0.042 ± 0.006
TVA {1; 300; 50}	890 ± 33	700 ± 3	219 ± 50	0.053 ± 0.009	0.076 ± 0.012	0.039 ± 0.005
TVA {1; 400; 50}	956 ± 44	793 ± 35	219 ± 10	0.066 ± 0.016	0.090 ± 0.035	0.053 ± 0.011
TVA {1; 400; 500}	931 ± 141	804 ± 43	217 ± 29	0.062 ± 0.008	0.076 ± 0.009	0.052 ± 0.003
TVA {1; 400; 1000}	971 ± 11	813 ± 10	258 ± 30	0.055 ± 0.008	0.075 ± 0.016	0.059 ± 0.004

## Data Availability

The original contributions presented in this study are included in the supplementary material. Further inquiries can be directed to the corresponding author.

## Supplementary Materials

The following supporting information can be downloaded at: https://www.mdpi.com/article/10.3390/ma18153603/s1, Figure S1: SEM images of the surface morphologies of samples treated by PEO without the addition of ADP; Figure S2: SEM image of the surface morphologies of samples treated by PEO with 5 wt.% ADP additives, a magnified view; Figure S3: Cross-section SEM images of samples treated by PEO with varying ADP concentration: (a) 0.1, (b) 0.5, (c) 1, (d) 2, and (e) 5 wt.%. All coatings were synthesized at 400 V and 50 Hz; Figure S4: Cross-section SEM images of samples treated by PEO under varying electrical parameters: (a) 200 V/50 Hz; (b) 300 V/50 Hz; (c) 400 V/50 Hz; (d) 400 V/500 Hz; (e) 400 V/1000 Hz. All coatings were synthesized in molten electrolyte with 1 wt.% ADP.

## Abbreviations

The following abbreviations are used in this manuscript:

PEO	Plasma electrolytic oxidation;
ADP	Ammonium dihydrogen phosphate;
SEM	Scanning electron microscopy;
HAp	Hydroxyl-apatite;
CA	Contact angle;
EDX	Energy-dispersive X-ray spectroscopy on SEM;
XRD	X-ray diffraction;
TVA	Titanium vanadium alloy substrate, Ti-6Al-4V;
RHE	Reference hydrogen electrode.
