# Supplementary material for: Development of PEO in Low-Temperature Ternary Nitrate Molten Salt on Ti6Al4V
Source: Materials (Basel). 2025 Jul 31;18(15):3603. doi: 10.3390/ma18153603 (PMC12348440; doi:10.3390/ma18153603)
Supplement: Supplementary file 1 [file materials-18-03603-s001.zip › materials-3757299-supplementary.pdf]

SUPPLEMENTARY MATERIALS

Development of PEO in low-temperature ternary nitrate molten salt on Ti6V4Al

Michael Garashchenko [1], Yuliy Yuferov [1] and Konstantin Borodianskiy [1,*]

[1] Department of Chemical Engineering, Ariel University, Ariel, Israel; mihail.garashch@msmail.ariel.ac.il; yuliyy@ariel.ac.il; konstantinb@ariel.ac.il
* Correspondence: konstantinb@ariel.ac.il; Tel.: +972-3-9143085

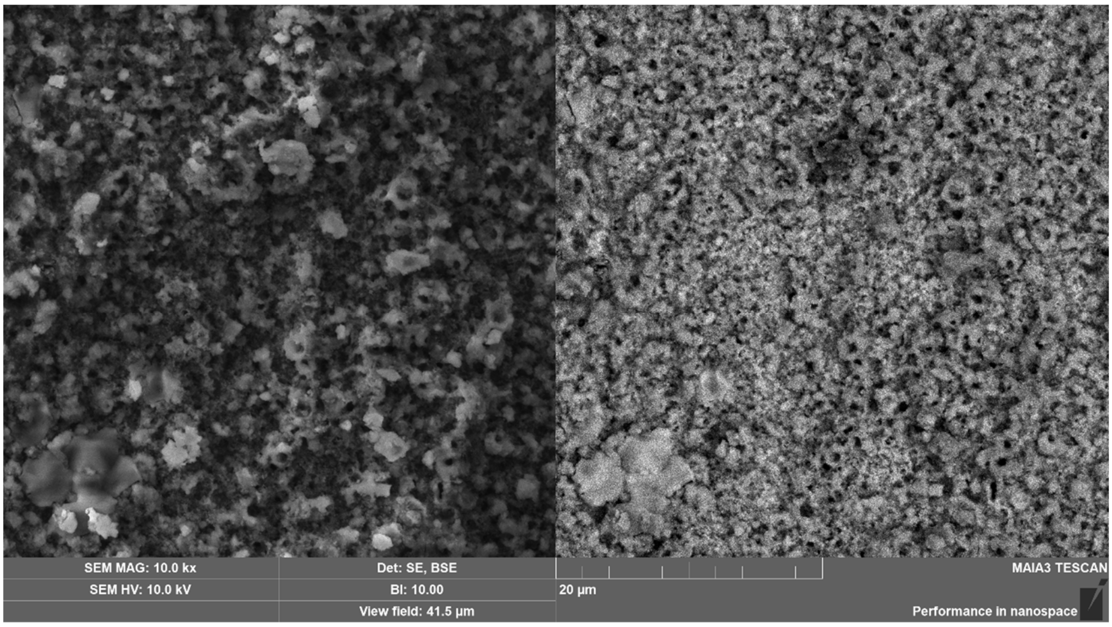

Figure S1 – SEM images of the surface morphologies of samples treated by PEO without the addition of ADP.

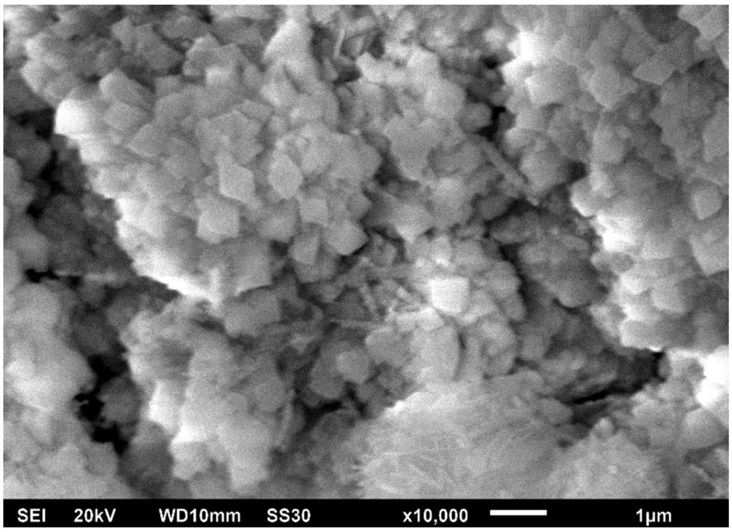

Figure S2 – SEM image of the surface morphologies of samples treated by PEO with 5 wt.% ADP additives, a magnified view.

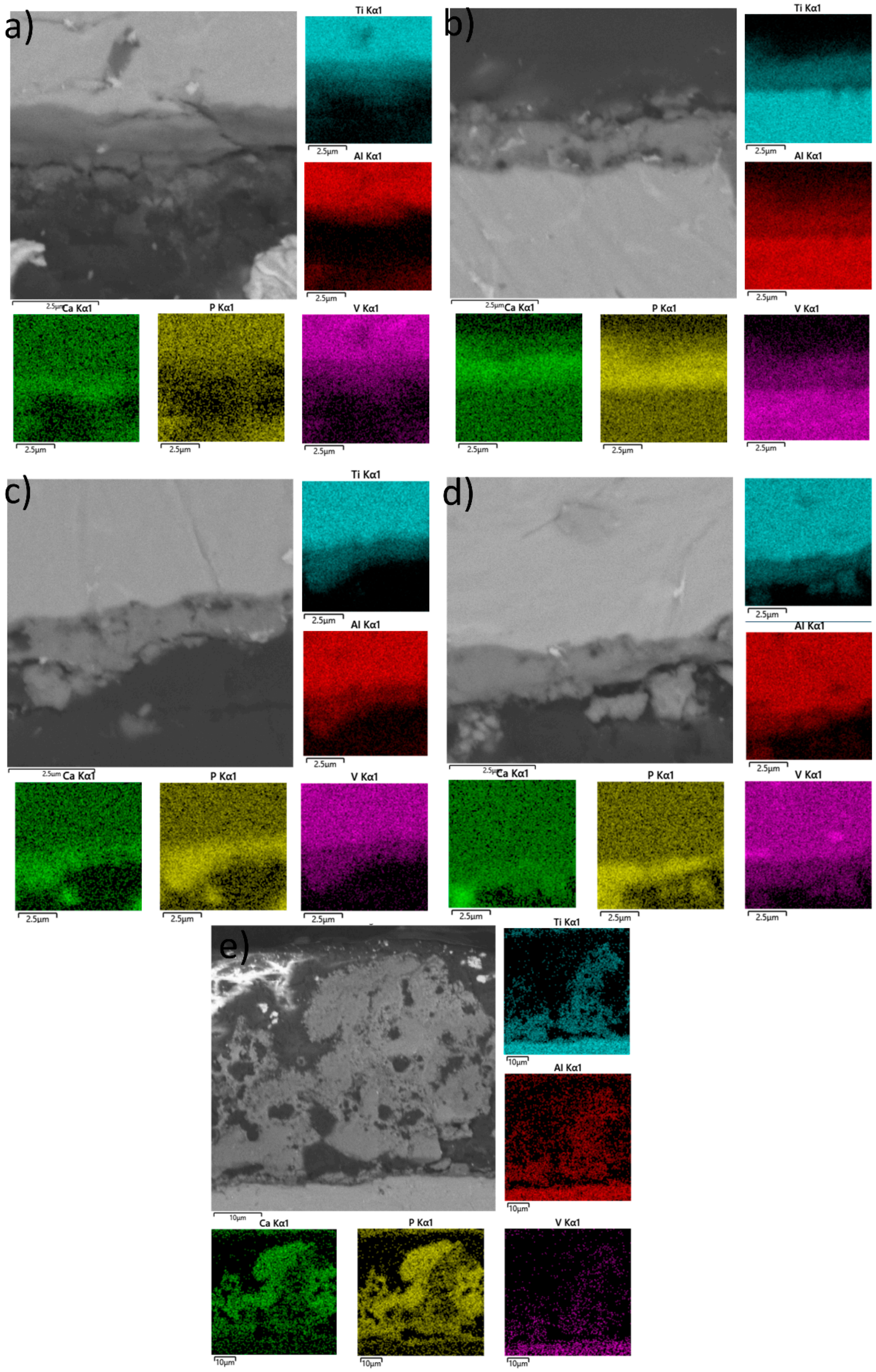

Figure S3 – Cross-section SEM images of samples treated by PEO with varying ADP concentration: a) 0.1, b) 0.5, c) 1, d) 2, and e) 5 wt.%. All coatings were synthesized at 400V and 50 Hz.

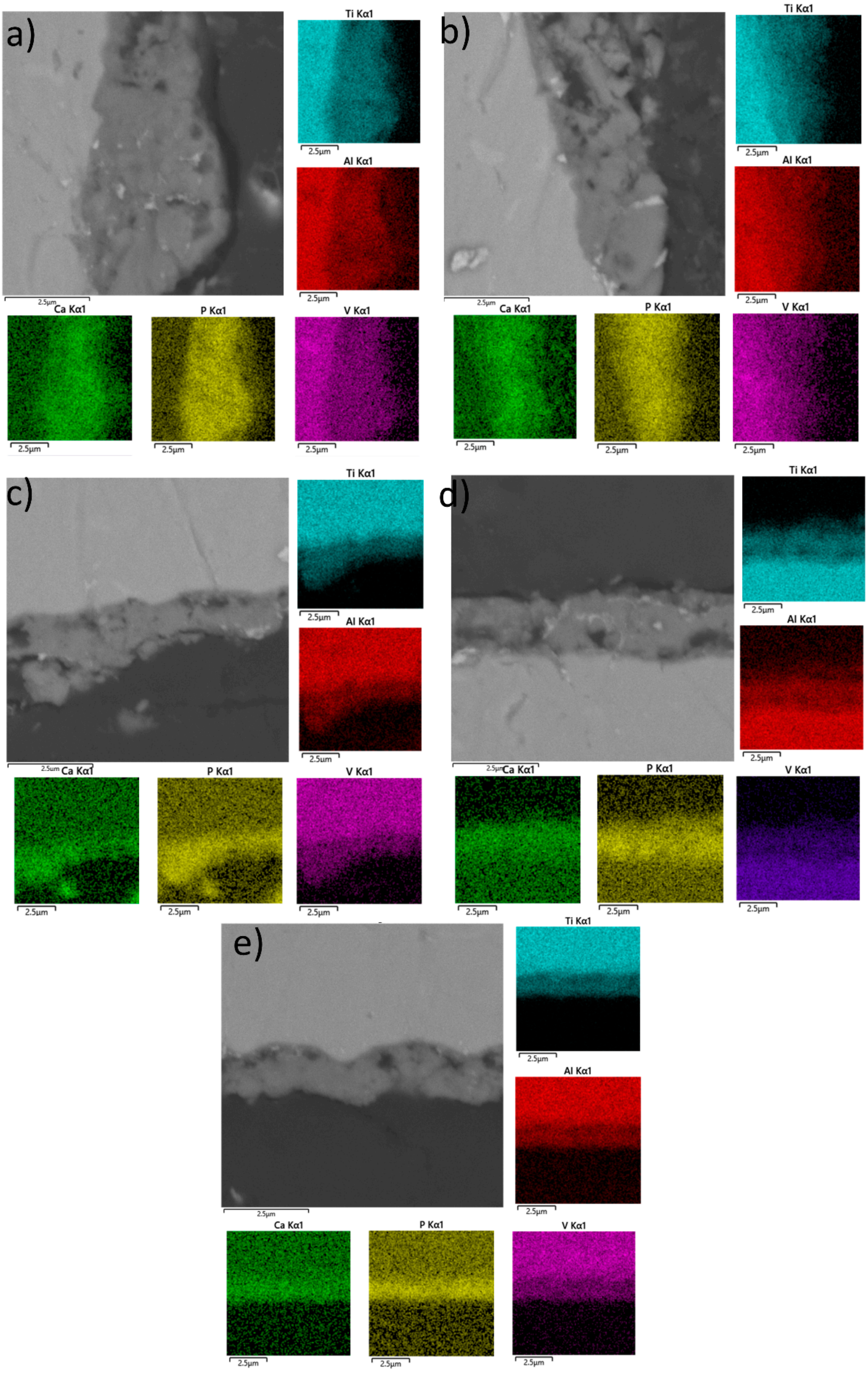

Figure S4 – Cross-section SEM images of samples treated by PEO under varying electrical parameters: a) 200V/50Hz; b) 300V/50Hz; c) 400V/50Hz; d) 400V/500Hz; e) 400V/1000Hz. All coatings were synthesized in molten electrolyte with 1 wt.% ADP.
